# 2379: Enhancing the clinical and translational enterprise through research staff development

**DOI:** 10.1017/cts.2017.176

**Published:** 2018-05-10

**Authors:** Stephanie A. Freel, Miranda West, Denise Snyder

**Affiliations:** Duke University, Durham, NC, USA

## Abstract

OBJECTIVES/SPECIFIC AIMS: Our objectives are to provide opportunities for graduate students, clinical interns, and postdoctoral fellows in traditional training programs to have immersive experiences in clinical research conduct from a CRP perspective. In addition, we aimed to address common causes of job dissatisfaction by providing professional development and networking opportunities for the existing CRP workforce. METHODS/STUDY POPULATION: In collaboration with the CTSA workforce development group, the Duke Office of Clinical Research hosted a site visit for 19 PhD scientists interested in nontraditional career pathways and a short lecture series on project management careers in clinical research. Additionally, we crafted specific clinical research training electives for 20 masters students and 10 dietetic interns. Finally, in collaboration with UNC-CH, we combined Research Professional Networks to provide a pilot joint professional development event for 109 CRPs from both schools. RESULTS/ANTICIPATED RESULTS: The number of Masters students enrolling in the CRP elective grew from 7 students in year 1 to 13 students currently enrolled. A retro-pre/postprogram adapted CRAI survey was issued following program completion. Students self-reported increases in competence across all 24 skills measured. Largest increases were seen in areas specific to CRP roles such as consenting patients, understanding the IRB, and reviewing key study documents. A baseline culture survey issued at the joint Duke/UNC CRP event garnered a 65% response rate and indicated that the principal gaps in professional training are in communications, teamwork, leadership, and professionalism. Moreover, respondents indicated that creating a sense of community and providing networking opportunities were the most important outcomes for future CRP collaborations. Future evaluations of both of these programs will allow us to tailor training to be most effective in strengthening our CRP workforce. DISCUSSION/SIGNIFICANCE OF IMPACT: These initiatives lay the groundwork for the development of a robust training pipeline into CRP careers. Future initiative will apply lessons learned toward creating internship programs aimed at improving diversity and inclusion within these careers. In addition, by addressing the professional development needs of the existing workforce, we create a sustainable environment for well-trained professionals. By evaluating these primary initiatives, we can better define the critical elements that must be included in CRP educational, development, and support programs and subsequently apply these to ultimately improve the clinical and translational research being conducted in academic research settings.

